# On the Medical Employment of Extracts of Flesh and Blood

**Published:** 1852-01

**Authors:** 


*On the Medical Employment of Extracts of Flesh and Blood.* By MM. Breslau and Mauthner.

Dr. Bauchner states that such great benefit has been derived from the employment of Dr. Breslau's *extractum carnis,* as a remedy in the diseases of exhaustion in children, that it ought to find a place in the materia medica. Fresh ox flesh, freed from fat, first finely chopped up, and then well beaten in a stone mortar, with a little cold or lukewarm distilled water, is afterward submitted to a good press. The cake is again similarly treated, and when the juice is thus pressed out of it, it may still, when seasoned, be advantageously employed as food. The juice, reddish in color, is immediately heated sufficiently to coagulate the albumen, and is then evaporated in a water bath to the ordinary consistency of an extract. As ordinary ox flesh contains only 1 in 1000 of kreatin, while that of the heart, according to Gregory, contains from 1-37 to 1-41, this is the part employed by Dr. Breslau at the chief apothecary establishment in Munich. The extract is of agreeable odor and taste, and is easily soluble in water, when it reddens litmus. By the addition of caramel to the juice, the taste and consistency of the extract is much improved.

In the exhausting diseases of children, Dr. Mauthner strongly recommends his *extractum sanguinis bovis.* Fresh blood caught from the slaughtered animal is passed through a sieve, and then evaporated in a water bath to dryness, rubbing it up into powder when cold. From ten to twenty grains are given per diem, in a little water, the solubility being increased by the addition of a few drops of spirit of wine. Dr. Mauthner has now employed it with great success in about twenty cases, several of which were reduced to an apparently desperate condition before commencing with it. Four cases are related as examples. 1. A girl, æt. 7, had suffered from catarrhal diarrhœa, during eight days, which completely reduced her. She took one scruple of the extract daily, from the 28th of August to the 10th of September, when she left quite well. 2. A girl, æt. 12, was reduced to a mere skeleton by diarrhœa; and after being treated by various means, and constantly getting worse, she commenced the extract on the 8th of September, and was quite cured by the 27th. 3. A child, æt. 7, very liable to scrofulous opthalmia, and now reduced to the lowest point by diarrhœa supervening on hip-joint disease, continued the extract from the 8th to the 22d of September, when he left the hospital cured as regards his immediate cause of exhaustion. 4. A child, æt. 4, suffering from hectic and manifesting broncophony, had a *fistula ani* form, and was reduced to a complete state of anæmia. He recovered by continuing the extract from the 1st to the 12th of September. It is by no means a disagreeable remedy, and a child will take it when it will not take or rejects ordinary medicines. It does not appear in the stools, scarcely a trace is found in the urine, and it is never vomited. It is especially useful in what Dr. Mauthner terms *exhaustio scrofulosa,* and the child will take it much better than the *ol. jecoris.* It is of no use in the acute marasmus and anæmia of young infants, due to bringing up by hand, who are brought to the hospital during the last days of their wretched existence. —*Buchner's Repert.:* No. 19, p. 90; *Journ. fur Kinderkrank,* xvi, p. 56.

